# A pathogen-derived small RNA modulates host immunity for chronic parasitism

**DOI:** 10.1080/21505594.2026.2757924

**Published:** 2026-11-09

**Authors:** Tongyao Liu, Quangping Li, Jieming Liu, Zhengming Qiang, Meichun Xiang, Wenjia Li, Xingzhong Liu

**Affiliations:** a State Key Laboratory of Medicinal Chemical Biology, Key Laboratory of Molecular Microbiology and Technology of the Ministry of Education, Department of Microbiology, Frontiers Science Center for Cell Responses, College of Life Science, Nankai University, Tianjin, China; b Key Laboratory of State Administration of Traditional Chinese Medicine, Sunshine Lake Pharma Co., Ltd., Dongguan, China; c State Key Laboratory of Mycology, Institute of Microbiology, Chinese Academy of Sciences, Beijing, China; d College of Life Science, University of Chinese Academy of Sciences, Beijing, China

**Keywords:** *Ophiocordyceps sinensis*, cross-kingdom regulation, milRNA (microRNA-like RNA), chronic infection, coevolution

## Abstract

Parasites that rely on prolonged host exploitation must balance immune evasion with host survival. The entomopathogenic fungus *Ophiocordyceps sinensis* maintains its insect host, *Thitarodes xiaojinensis*, for months before host death, but the mechanisms stabilizing host immunity during this period are unclear. Here, we identify a cross-kingdom regulatory strategy mediated by a fungal microRNA-like RNA (milRNA), Os-milRNA2, which is delivered into host immune tissues and directly targets the antimicrobial peptide gene *attacin-1*. Os-milRNA2 accumulates during mid-stage infection, repressing immune gene expression, whereas its inhibition restores host immunity and restricts fungal proliferation. Comparative analyses reveal lineage-specific retention of the Os-milRNA2 binding site in the *T. xiaojinensis* 3’UTR of *attacin-1*, indicating regulatory coevolution. Our findings show that a slow-killing parasite attenuates specific immune effectors to maintain host-pathogen homeostasis during chronic infection.

## Graphical abstract

**Figure:**
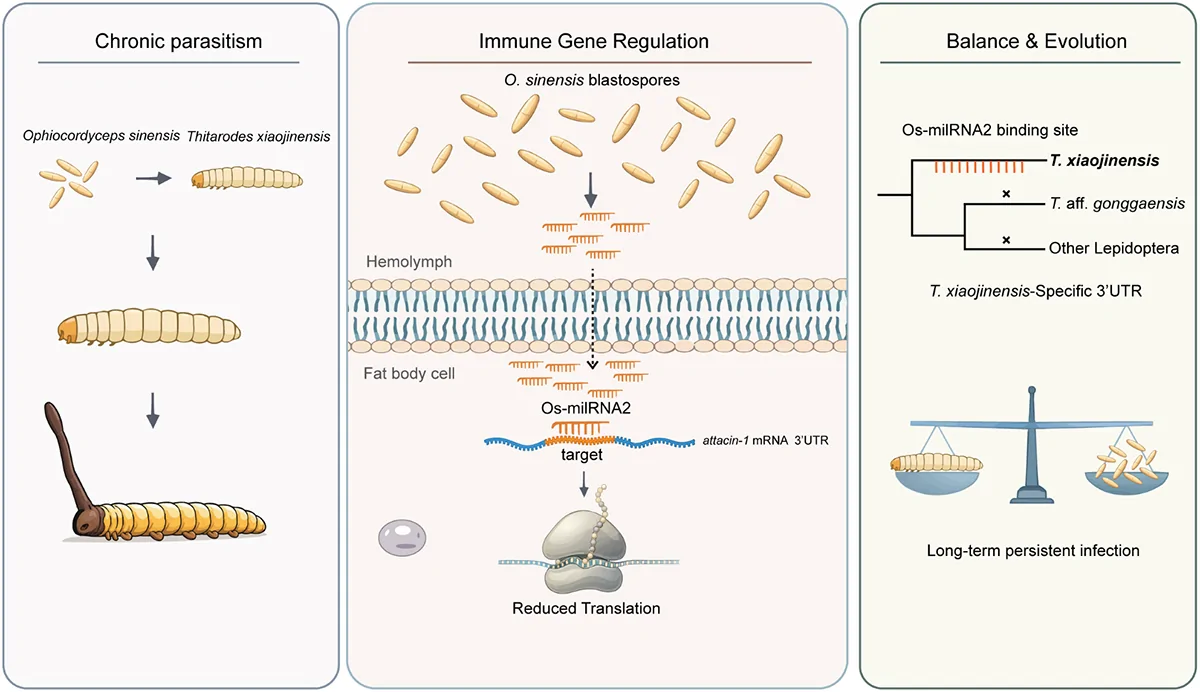

## Introduction

Persistent parasitism poses a fundamental evolutionary dilemma: parasites must extract resources from their hosts while preserving host viability long enough to ensure their own transmission. For pathogens that establish chronic infections, successful parasitism depends not on eliminating host immunity, but on precisely tuning it [1,2]. This requirement places immune regulation at the center of host–parasite coevolution, driving reciprocal adaptation between host defense systems and parasite immune-modulatory strategies.

Entomopathogenic fungi provide powerful models for examining these dynamics. Unlike acute pathogens that rapidly overwhelm host defenses, some fungal parasites sustain long-term infections within living hosts, maintaining a dynamic equilibrium between proliferation and immune containment [3,4]. The fungus *Ophiocordyceps sinensis*, the etiological agent of a highly valued traditional medicine Chinese cordyceps characterized by its complex metabolic profiling and diverse pharmacological potential [5–7], exemplifies this strategy by maintaining its insect host, *Thitarodes xiaojinensis*, across multiple larval instars before ultimately inducing host death for reproduction [8]. The infection of *Thitarodes* spp. larvae by *O. sinensis* follows a prolonged process with a clear physiological turning point [8–10]. After initial colonization (6 dpi), the fungus undergoes massive asexual proliferation as blastospores in the host hemolymph (30 dpi). A critical threshold is reached at 90 dpi, representing a phase of stable coexistence where fungal load peaks. Beyond this 90-day mark, the fungus begins complex morphological differentiation (150–180 dpi), signaling the transition from chronic infection to host systemic collapse and eventual mummification. This 90-day juncture serves as a pivotal regulatory node, marking the shift in the balance between fungal virulence and host immunity. This obligate parasitic specificity is further underpinned by molecular co-adaptation at the gene-expression level, as evidenced by the synchronized codon usage bias between the genomes of the fungus and its specific host [11]. During this extended parasitic phase, the fungus proliferates within the hemolymph as blastospores without triggering catastrophic immune failure, indicating that host immunity is actively modulated rather than suppressed. Transcriptomic studies [12–14] showing fluctuating antimicrobial peptide (AMP) expression during infection further suggest that immune homeostasis is a key target of parasite manipulation, yet the mechanisms enabling such fine control remain largely unresolved.

At the molecular level, host–parasite coevolution is often characterized by Red Queen dynamics [15–18], manifesting as a reciprocal arms race where host immune adaptations are met by counter-adaptations in parasite virulence mechanisms [19–21]. In insects, humoral immunity relies heavily on AMP production *via* conserved signaling pathways such as Toll and Imd [22,23], making AMP genes and their regulatory regions recurrent targets of pathogen-mediated selection. While proteinaceous effectors have long been viewed as the principal tools of immune suppression, recent work has revealed pathogen-derived small RNAs (sRNAs) as an alternative and evolutionarily agile class of virulence factors [24–27]. Acting through sequence complementarity, sRNAs can silence host genes without eliciting immune recognition, but this same specificity renders sRNA–target interactions highly sensitive to sequence change. As a result, sRNAs are expected to drive rapid, reciprocal evolution between pathogen effector sequences and host regulatory elements.

In fungi, small RNAs are termed microRNA-like RNAs (milRNAs) because they share functional similarities with canonical microRNAs while often utilizing distinct biogenesis mechanisms [28]. In this study, we investigated the molecular basis of immune stabilization during the chronic infection of *O. sinensis*. We identified a fungal microRNA-like RNA, Os-milRNA2, that serves as a key regulator of host immunity. Our results show that *O. sinensis* exploits this evolutionary vulnerability by deploying Os-milRNA2 to fine-tune host immunity during chronic infection. Os-milRNA2 accumulates within host immune tissues (fat body [29]) and targets the 3’ untranslated region (3’UTR) of the AMP gene *attacin-1*, dampening antimicrobial responses without compromising host survival. Functional interference of Os-milRNA2 restores immune activity and restricts fungal proliferation, establishing this sRNA as a key virulence determinant. Importantly, comparative evolutionary analyses reveal a hallmark of coevolution at this interface: while the *attacin-1* coding sequence remains highly conserved across Lepidoptera, its 3’UTR exhibits apparent lineage-specific divergence.

The Os-milRNA2 binding site is specifically identified in the natural host *T. xiaojinensis*, representing a lineage-specific co-adaptation, but is absent in non-host species and the closely related *T*. aff. *gonggaensis*. These findings identify cross-kingdom sRNA targeting as a powerful driver of regulatory sequence evolution and reveal how chronic parasites fine-tune, rather than extinguish host immunity through coevolved RNA–RNA interactions.

## Results

### Fungal microRNA-like RNAs accumulate in host immune tissues during infection

To investigate whether *O. sinensis* delivers small RNAs into host tissues during infection, we profiled fungal-derived sRNAs in the fat body of infected *T. xiaojinensis* larvae. Fat bodies were carefully dissected and rinsed three times with Ringer’s solution to remove hemolymph. To rigorously confirm the absence of fungal cells in the fat bodies and avoid potential false negatives, tissues were thoroughly homogenized using a cryo-grinder with beads to ensure complete lysis of any potential fungal residues. The absence of fungal colonization was confirmed by Reverse transcription-quantitative polymerase chain reaction (RT-qPCR) targeting the *O. sinensis* F-actin capping protein subunit beta gene (Os*-FcpB*). While this fungal marker was robustly amplified in whole infected larvae (positive control), it remained undetectable in all purified fat body samples (Figure 1(A,B)).

**Figure 1.**
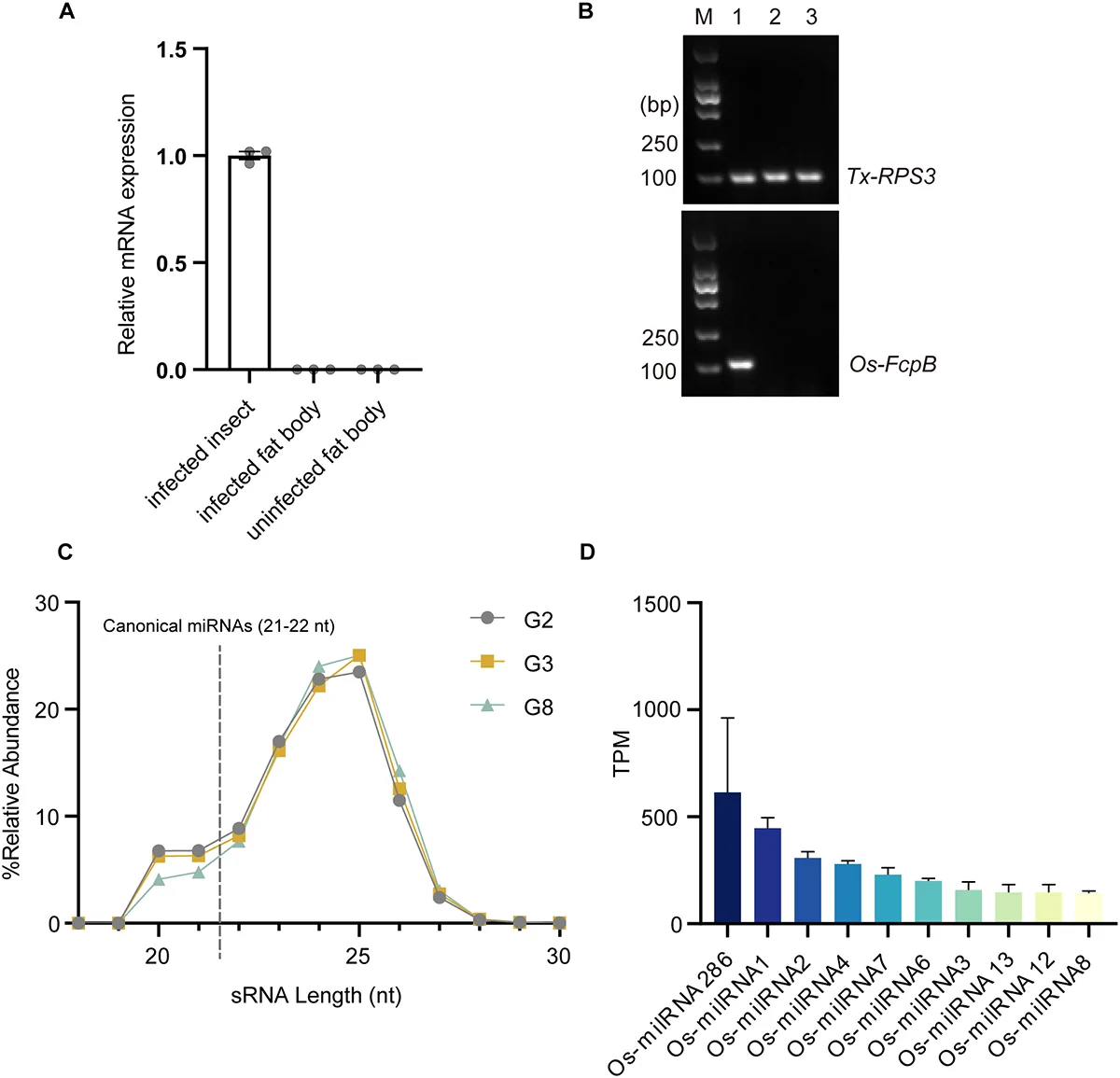
Identification of *O. sinensis*-derived milRNAs in *T. xiaojinensis*. (A) Quantitative PCR (qPCR) analysis of the fungal F-actin capping protein beta subunit gene (*Os-FcpB*) expression in *T. xiaojinensis* fat body samples to verify the absence of fungal contamination. Expression was normalized to the host reference gene, *tx-RPS3*. The whole infected insect served as a positive control. (B) RT-PCR analysis further confirming sample purity. The host gene *tx-RPS3* was detected in all samples, whereas the fungal gene *Os-FcpB* was only detected in the positive control (lane 1). Lane M: DNA marker; lane 1: infected insect; lane 2: infected fat body; lane 3: uninfected fat body. (C) Length distribution of small RNAs (sRNAs) mapped to the *O. sinensis* genome. G2, G3, and G8 represent three biological replicates. The y-axis represents the %relative abundance (percentage of mapped reads). (D) The ten most abundant *O. sinensis* milRNAs identified in *T. xiaojinensis*, ranked by expression level in transcripts per million (TPM). Data are presented as mean ± SEM from three biological replicates.

Total RNA was extracted and subjected to Illumina NovaSeq 6000 sequencing. Across three biological replicates, we obtained 29.7, 32.3 and 38.6 million clean sRNA reads per sample. Reads of 18 – 30 nucleotides were retained and mapped to the genome of *O. sinensis*, with 1.91%–5.86% of reads mapping uniquely (Table 1). After removal of tRNA and rRNA sequences, the remaining reads were considered candidate fungal microRNA-like RNAs (milRNAs). These fungal-derived sRNAs displayed a distinctive size distribution, with a strong enrichment at 25 nucleotides (Figure 1(C)), consistent with previously reported *O. sinensis* milRNAs and distinct from canonical plant and animal miRNAs (21–22 nt) [30]. In total, 709,513, 401,144, and 1,291,437 fungal sRNA reads were identified across replicates. The ten most abundant milRNAs are shown in Figure 1(D). Bioinformatic analysis confirmed their exogenous fungal origin, as these sequences mapped perfectly to the *O. sinensis* genome but lacked endogenous templates in the *T. xiaojinensis* genome (Supplementary Table S2). Three highly abundant milRNAs with defined genomic loci were selected for downstream analysis (Table 2). Together, these results demonstrate that *O. sinensis* milRNAs accumulate within host immune tissues during chronic infection.

**Table 1.** sRNA mapped to *O. sinensis* genome.

Sample ID	G2	G3	G8
reads processed	28,862,687	21,052,696	22,047,770
reads with at least one reported alignment	709,513 (2.46%)	401,144 (1.91%)	1,291,437 (5.86%)
reads that failed to align	27,628,596 (95.72%)	20,461,707 (97.19%)	20,525,806 (93.10%)
reads with alignments suppressed due to -m^a^	524,578 (1.82%)	189,845 (0.90%)	230,527 (1.05%)
Reported alignments	709,513	401,144	1,291,437

a:-m = 1, limit the maximum number of alignments = 1

**Table 2.** Top3 milRNAs in *O. sinensis* genome.

	Locus^b^	Strand	Sequence	Length
OsmilRNA286	Chr6:1495437-1495461	+	TGACTGCTGGAATATCGAAATCCGC	25
OsmilRNA1	Chr3:215222-215245	+	TTCGGGACGTGTCACCTATTCTGG	24
OsmilRNA2	Chr3:214832-214855	+	TAATACGTTAGACGTCTCCGATGG	24

^b^Position according to the reference genome of *O. sinensis* (GWHGDWT00000000.1).

### Predicted targets of fungal milRNAs are enriched in immune and metabolic pathways

To assess the potential functional impact of fungal milRNAs on host physiology, we predicted host target genes of the three most abundant milRNAs: Os-milRNA286, Os-milRNA1, and Os-milRNA2, using three independent software (miRanda, RNAhybrid, and PITA). Intersection analysis identified 351, 384, and 79 high-confidence targets, respectively (Figure 2(A,D,G)).

**Figure 2.**
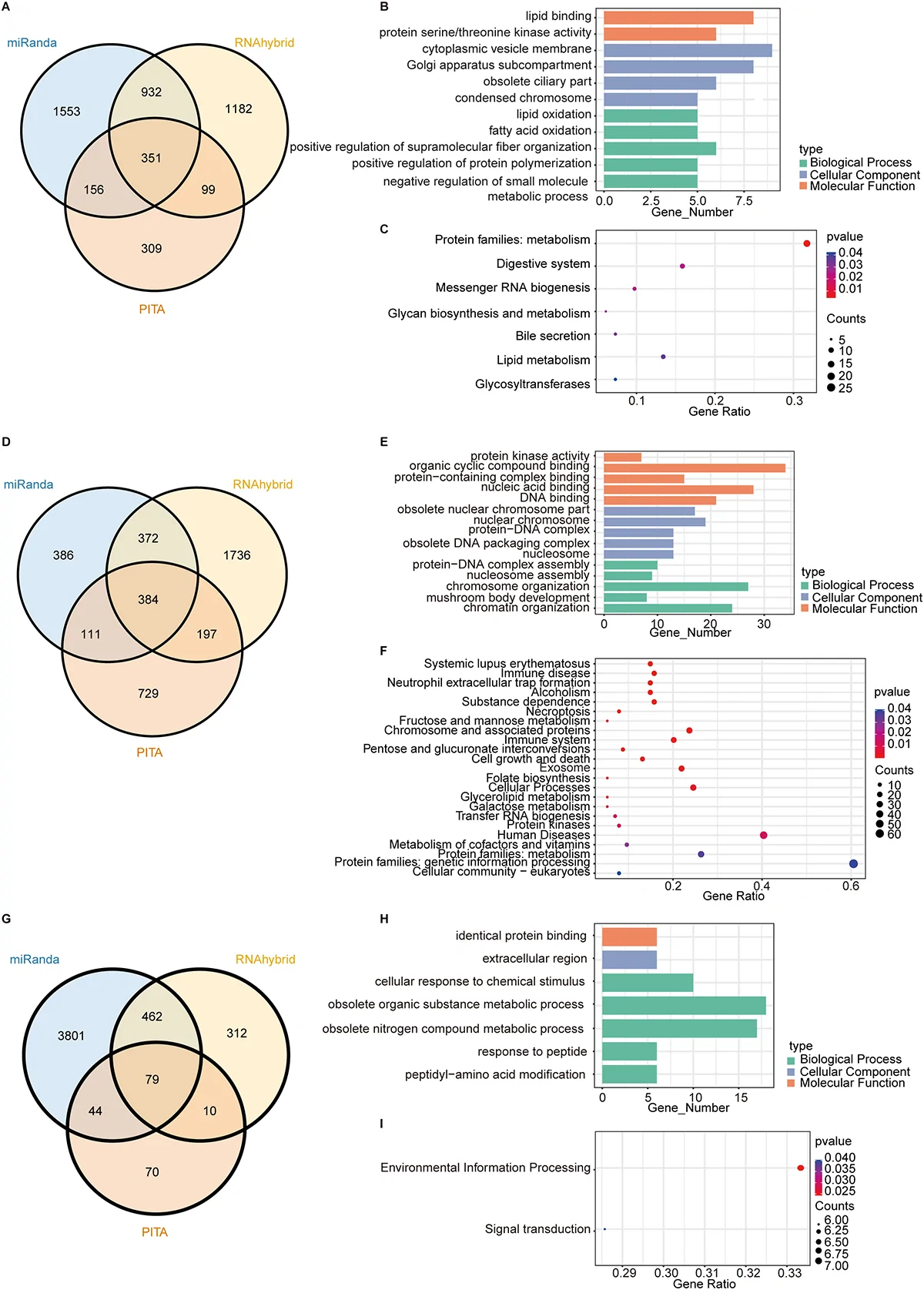
Prediction and functional enrichment analysis of target genes for the three most abundant *O. sinensis* milRNAs in *T. xiaojinensis*. Results of target prediction, gene Ontology (GO) enrichment, and Kyoto encyclopedia of genes and genomes (KEGG) pathway analysis are shown for three distinct milRNAs. (A-C) analysis for Os-milRNA286. (A) Venn diagram illustrating the overlap of target genes predicted by miRanda, RNAhybrid, and pita, identifying 351 common targets. (B) GO enrichment analysis of the 351 common target genes. Bars are colored by functional category: biological process (green), cellular component (blue), and molecular function (orange). (C) KEGG pathway enrichment analysis of the 351 common targets, where dot color indicates the *p*-value and dot size represents the number of enriched genes (counts). (D-F) Analysis for Os-milRNA1. (D) Venn diagram of its predicted targets, identifying 384 common genes. (E) GO enrichment analysis and (F) KEGG pathway analysis of the 384 common targets. (G-I) Analysis for Os-milRNA2. (G) Venn diagram of its predicted targets, identifying 79 common genes. (H) GO enrichment analysis and (I) KEGG pathway analysis of the 79 common targets.

Gene Ontology analysis revealed distinct functional biases among the milRNAs. Os-milRNA286 targets were significantly enriched in lipid metabolism related processes, including lipid binding and fatty acid oxidation (Figure 2(B)). Os-milRNA1 targets were enriched in chromatin organization and nucleosome assembly, as well as immune-related terms such as defense response and innate immunity (Figure 2(E)). Os-milRNA2 targets were enriched in extracellular and stimulus-response categories (Figure 2(H)).

KEGG pathway analysis further supported these distinctions. Os-milRNA286 targets were associated with lipid metabolism pathways (Figure 2(C)), whereas Os-milRNA1 targets were enriched in immune and inflammation-related pathways, including systemic lupus erythematosus (represented by histone genes in *T. xiaojinensis*, Supplementary Table S3), neutrophil extracellular trap formation (analogous to hemocyte extracellular traps/Etosis in insects), and necroptosis (Figure 2(F)). Os-milRNA2 targets were enriched in signaling and environmental information processing pathways (Figure 2(I)).

Notably, Os-milRNA2 was predicted to target the antimicrobial peptide gene *attacin-1*, a key effector of insect humoral immunity (Supplementary Table S4). Given our focus on immune homeostasis during chronic infection, we prioritized the Os-milRNA2–*attacin-1* interaction for further investigation.

### Os-milRNA2 directly targets the 3’UTR of the antimicrobial peptide gene attacin-1

To determine whether Os-milRNA2 directly regulates *attacin-1* expression, we performed dual-luciferase reporter assays in HEK293T cells. Approximately 400 bp of the *attacin-1* 3’UTRs containing the predicted target sites were cloned downstream of the firefly luciferase gene. Corresponding mutant constructs lacking the seed-matching sequence served as controls (Figure 3(A)). Co-transfection of Os-milRNA2 mimics significantly reduced luciferase activity driven by the wild-type *attacin-1* 3’UTRs, whereas mutation of the target sites abolished repression (Figure 3(B)). These results confirm that Os-milRNA2 specifically targets the 3’UTR of *attacin-1*.

**Figure 3.**
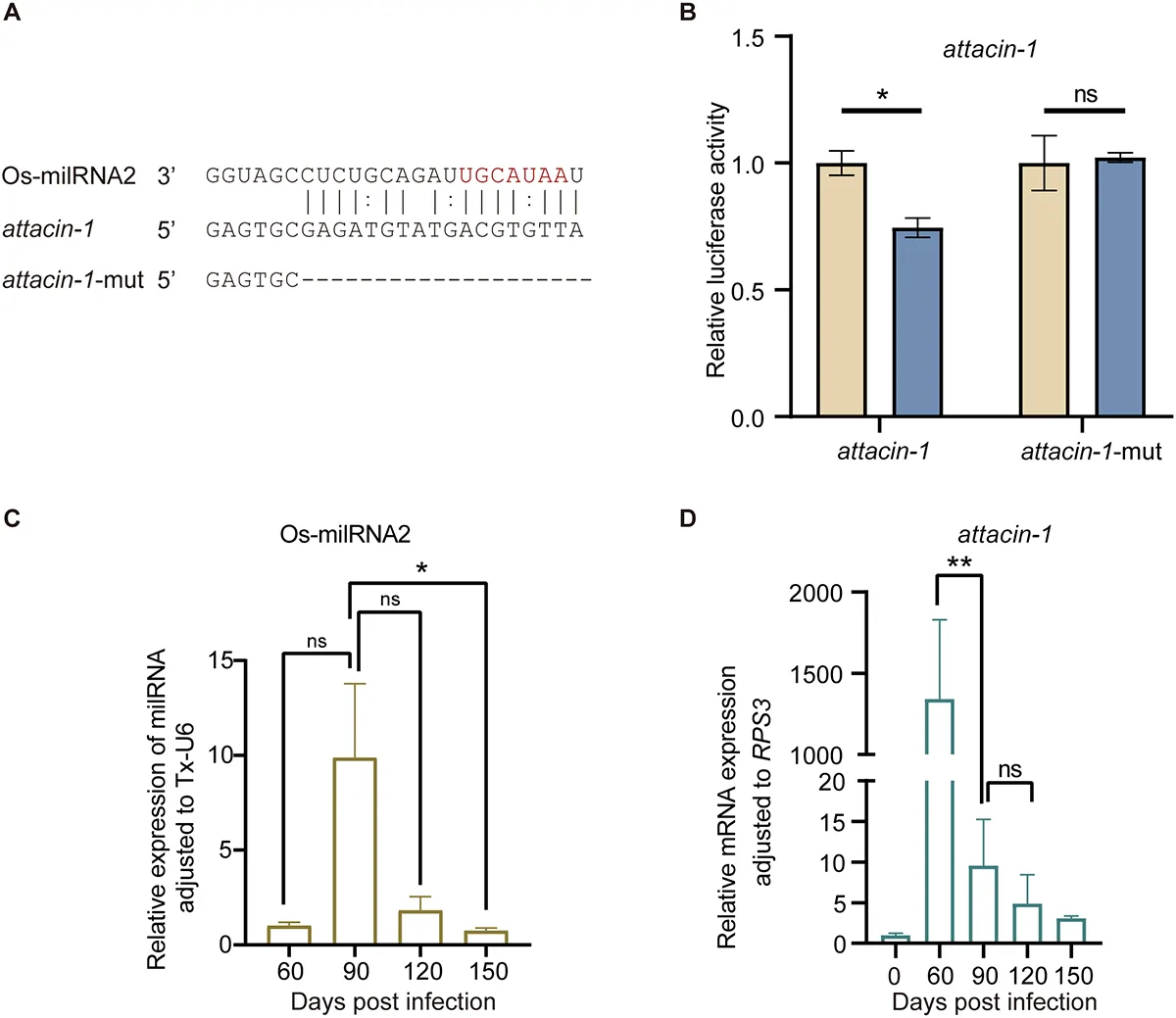
Functional validation of *T. xiaojinensis attacin-1* as direct targets of Os-milRNA2. (A) Schematic of the predicted binding sites for Os-milRNA2 within the 3’ untranslated regions (3’UTRs) of *T. xiaojinensis attacin-1*. The base pairing between Os-milRNA2 and the wild-type (wt) sequences is shown, along with the sequences carrying mutations (mut) in the seed region. (B) Dual-luciferase reporter assays performed in HEK293T cells. Co-transfection of Os-milRNA2 mimics with a reporter vector containing the wt 3’UTR of *attacin-1* resulted in a significant reduction in luciferase activity. This suppression was abolished when vectors with the mut 3’UTR sequences were used. (C-D) Temporal expression profiles at different time points post-fungal infection. Relative expression levels of (C) Os-milRNA2 and (D) Host *attacin-1*. Data are presented as mean ± SEM from three independent experiments. Statistical significance is indicated as follows: *p* < 0.05 (*), *p* < 0.01 (**), *p* < 0.001 (***); ns, not significant.

Consistent with a functional regulatory role in vivo, Os-milRNA2 abundance peaked at 90 days post-infection, coinciding with maximal fungal burden (Figure 3(C)). In contrast, *attacin-1* transcripts showed an inverse expression pattern, declining sharply at this stage (Figure 3(D)). These opposing dynamics support a role for Os-milRNA2 in modulating host immune responses during the mid-stage of chronic infection.

### Fungal Os-milRNA2 enters host fat body cells and suppresses attacin-1 expression

To directly test the activity of Os-milRNA2 in vivo, Cy3-labeled synthetic Os-milRNA2 was injected into the hemocoel of infected larvae at 90 days post-infection (Figure 4(A,B)). Injection of Os-milRNA2 significantly reduced *attacin-1* expression compared with Ringer’s solution controls (Figure 4(C)), confirming its inhibitory effect on host immunity.

**Figure 4.**
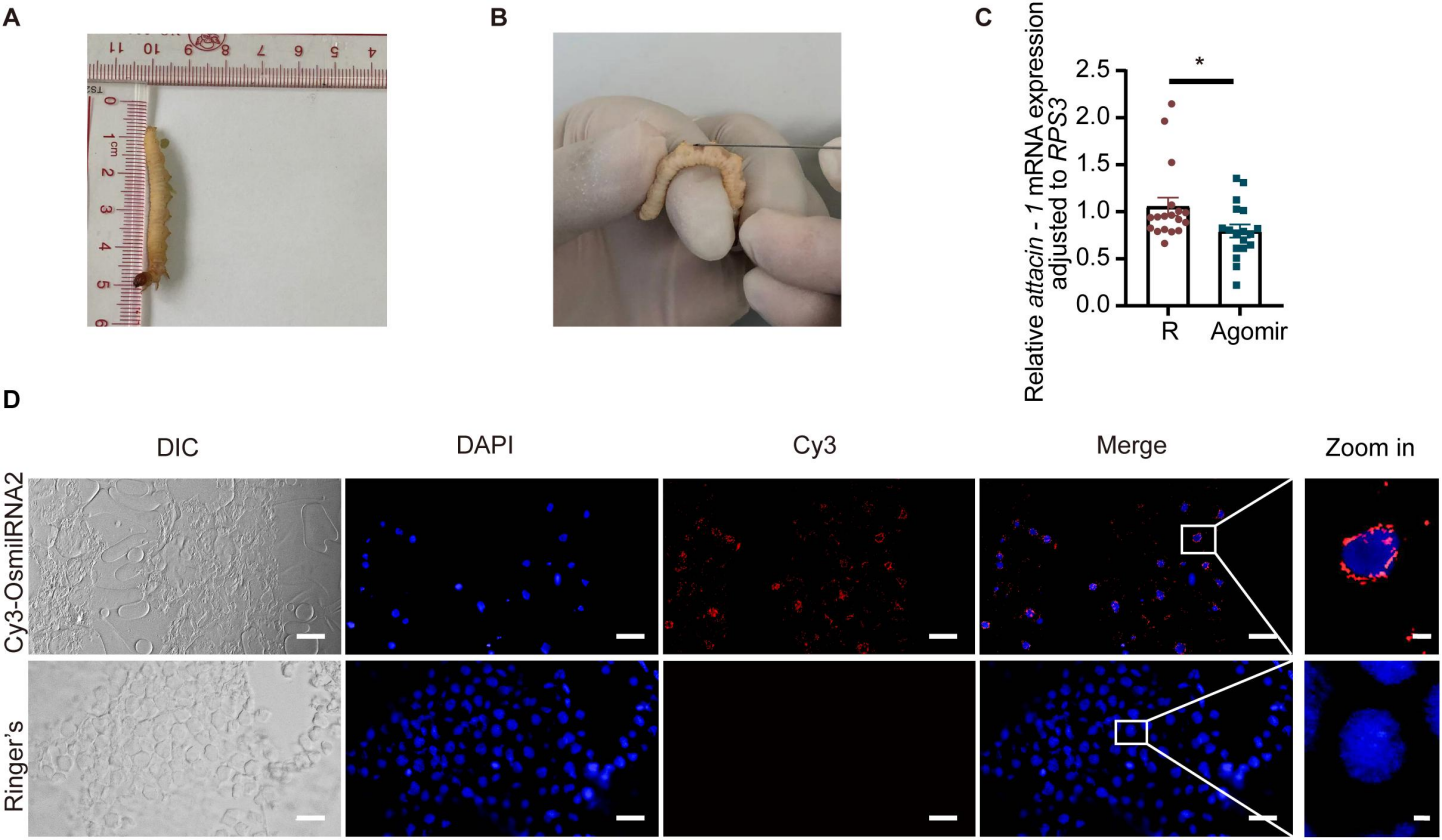
Os-milRNA2 suppresses *attacin-1* expression and translocates into fat body cells of *T. xiaojinensis* in vivo. (A) A *T. xiaojinensis* larva used for the in vivo experiments. (B) Schematic of the experimental procedure, illustrating the injection of Os-milRNA2 mimics into the larval hemocoel. (C) Relative mRNA expression of *attacin-1* in the fat body, quantified by qPCR at 24 hours post-injection with either Os-milRNA2 mimics or a negative control (NC). The results demonstrate significant downregulation of *attacin-1* by Os-milRNA2. (D) Fluorescence microscopy imaging showing the translocation of Os-milRNA2 into fat body cells. Cy3-labeled Os-milRNA2 (red) was injected into the hemocoel, and the fat body was dissected 24 hours later. Nuclei were counterstained with DAPI (blue). The merged image confirms the presence of Os-milRNA2 in the cytoplasm of fat body cells. Data are presented as mean ± SEM. Statistical significance: *p* < 0.01 (*). Scale bars, 50 μm. scale bars in zoom in: 5 μm.

Fluorescence microscopy revealed strong Cy3 signals within fat body cells 24 hours after injection, predominantly localized in the cytoplasm (Figure 4(D)). These observations demonstrate that fungal milRNAs can efficiently enter host immune cells and directly modulate antimicrobial gene expression in vivo.

### Inhibition of Os-milRNA2 restores host immunity and restricts fungal proliferation

To test whether Os-milRNA2 is required for fungal persistence, we performed in vivo loss-of-function experiments using an Os-milRNA2-specific antagomir. Infected larvae were injected weekly for 4 weeks with either an Os-milRNA2-specific antagomir, a non-targeting antagomir sequence (negative control, NC), or Ringer’s solution (Figure 5(A)). Os-milRNA2-specific antagomir treatment significantly increased the transcript abundance of *attacin-1* expression relative to Ringer’s and showed an increasing trend compared to the NC group, suggesting a relief of Os-milRNA2-mediated suppression (Figure 5(B)).

**Figure 5.**
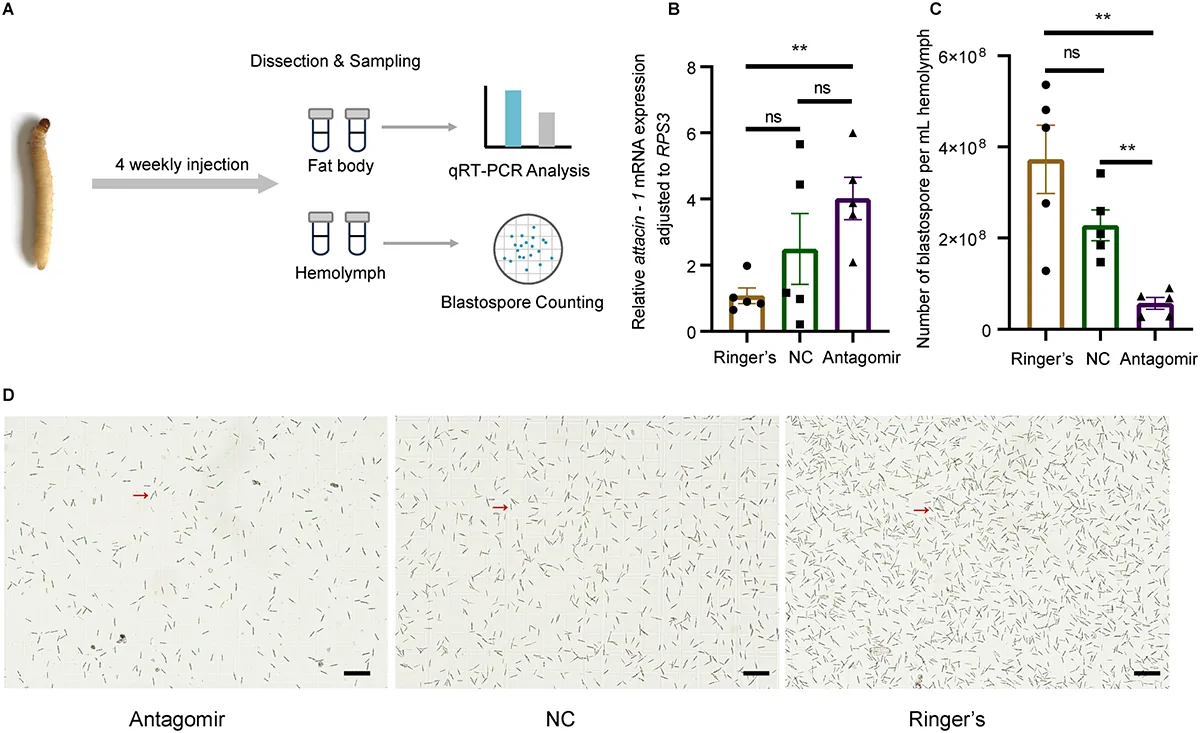
In vivo inhibition of Os-milRNA2 suppresses fungal proliferation in the host hemolymph. (A) Schematic illustration of the experimental workflow. *O. sinensis*-infected larvae received four weekly injections of either an Os-milRNA2 inhibitor (Antagomir), non-targeting antagomir sequence (NC) or Ringer’s solution. A scrambled negative control (NC) with identical chemical modifications was used to assess nonspecific effects. Fat body and hemolymph samples were collected 7 days after the final injection. (B) QRT-PCR analysis of *attacin-1* mRNA expression in the fat body, normalized to the reference gene *RPS3*. (C) Quantification of *O. sinensis* blastospores per mL of hemolymph harvested from treated and control larvae. (D) Representative microscopic images of host hemolymph visualized on a hemocytometer. Red arrows indicate *O. sinensis* blastospores. Samples were diluted 20-fold prior to observation. Scale bars = 100 µm. Data in (B) and (C) are presented as mean ± SEM. Each dot represents an individual larva (*n* = 5 per group). Statistical significance was determined by an unpaired two-tailed Student’s t-test. ns, not significant; **, *p* < 0.01.

Quantification of blastospores revealed a marked reduction in fungal load in Os-milRNA2-specific antagomir-treated larvae compared to the high blastospore counts observed in both the Ringer’s and NC groups (Figure 5(C)). This quantitative result was further corroborated by microscopic examination of hemolymph smears (Figure 5(D)). While the hemolymph from control larvae was densely populated with proliferating blastospores, the samples from Os-milRNA2-specific antagomir group exhibited a distinctively clear field with significantly fewer visible fungal cells. Together, these results demonstrate that Os-milRNA2 promotes fungal colonization by suppressing host *attacin-1* expression and that inhibiting this single fungal sRNA is sufficient to restore host immunity and limit *O. sinensis* proliferation in vivo.

### T. Xiaojinensis-specific target site divergence reveals a molecular arms race

To investigate the evolutionary dynamics underlying the interaction between Os-milRNA2 and its target sequence, we performed a comparative evolutionary analysis of *attacin* orthologs across representative insect species. We specifically restricted our dataset to the order Lepidoptera, including the ancient lineage *Thitarodes xiaojinensis* (host), *T*. aff. *gonggaensis* (host) and diverse non-host Ditrysian species such as *Bombyx mori* and *Ostrinia furnacali*. Binding affinity prediction analysis revealed an apparent evolutionary gradient in host specificity. Os-milRNA2 exhibited high-affinity binding to the *T. xiaojinensis attacin-1* 3’UTR (ΔG = −25.3 kcal/mol), whereas all tested non-host orthologs showed significantly weaker binding potential, with minimum free energies ranging from −6.4 to −12.6 kcal/mol (Figure 6(A), Supplementary Table S5). This substantial thermodynamic gap underscores the specialized adaptation of Os-milRNA2 to its natural host, suggesting that functional regulation is biologically restricted to the *T. xiaojinensis* lineage.

**Figure 6.**
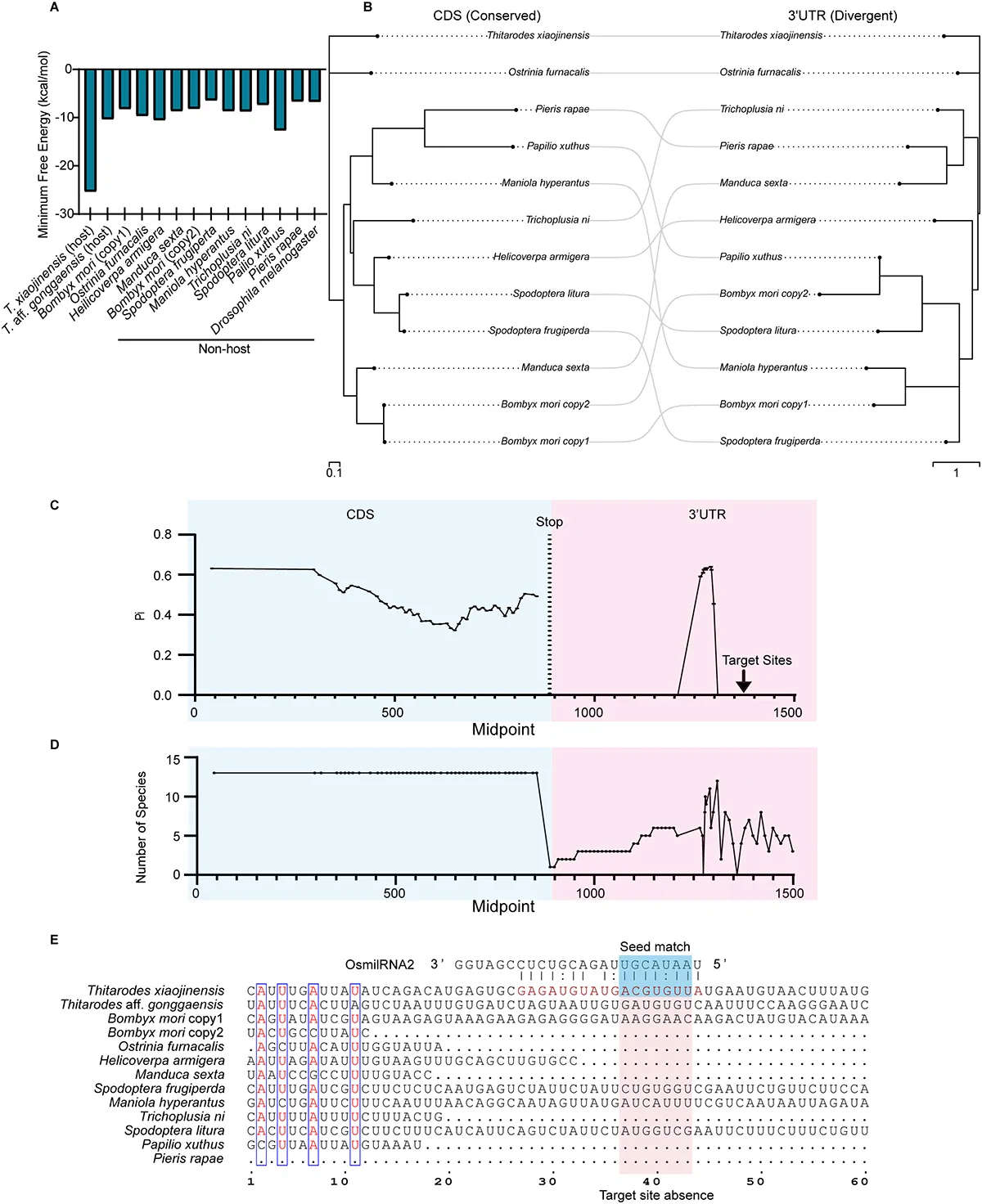
Evolutionary divergence and *T. xiaojinensis*-specific retention of the Os-milRNA2 target site. (A) Thermodynamic specificity of Os-milRNA2 targeting by miRanda. Bar graph showing the predicted minimum free energy (ΔG) of Os-milRNA2 binding to the 3’UTRs of *attacin* orthologs across representative lepidopteran species. While a high-affinity interaction (−25.3 kcal/mol) is predicted for the host *Thitarodes xiaojinensis* (highlighted in teal), no binding sites (N.D., not detected) were found in non-host insects, indicating strict host specificity. (B) tanglegram comparison of *attacin* orthologs coding (CDS) vs 3’UTR phylogenies. Left: CDS tree (GTR+G+I model); right: 3’UTR tree (T92+G model). Both trees were constructed using the maximum likelihood method with 1000 bootstrap replications. Grey lines connect the same species between the two trees. The CDS phylogeny reflects expected taxonomic relationships with short branch lengths (scale bar = 0.1), indicating functional conservation. The 3’UTR phylogeny displays a discordant topology (evidenced by extensive crossing lines) and significantly longer branch lengths (scale bar = 1). (C) Nucleotide diversity (π) analysis across the attacin orthologs 3’UTR loci. Sliding window analysis (window: 50 bp, step: 10 bp) reveals contrasting evolutionary constraints. The CDS region (shaded green) maintains a baseline diversity consistent with purifying selection. The 3’UTR exhibits a sharp peak of nucleotide diversity (π >0.6) in the region immediately flanking the Os-milRNA2 target site. Notably, the π value drops abruptly at the exact seed match location (position ~1371, indicated by the black arrow), corresponding to the extensive deletions and alignment gaps observed in non-host species (as shown in D). Dark gray bars along the x-axis indicate regions with extensive alignment gaps or unmapped sequences in non-host species, representing the deletion of the target site and downstream elements. (D) Alignment coverage representing the number of insect species present at each nucleotide position. (E) Molecular evidence of target site evasion. Multiple sequence alignment of the 3’UTR of *attacin* orthologs region flanking the target site. Blue boxes indicate conserved upstream motifs, confirming gene orthology. *T. xiaojinensis* sequence retains a perfect complementarity to the miRNA seed region (highlighted in green), whereas *T*. aff. *gonggaensis* and non-host species exhibit an absence of the target site (shaded in pink), termed “Target site absence”.

Phylogenetic analysis revealed contrasting evolutionary patterns between coding and regulatory regions. The *attacin* orthologs coding sequence was highly conserved and followed a canonical phylogenetic topology, whereas the 3’UTR showed extensive divergence and topological incongruence (Figure 6(B)). Sliding-window analyses of nucleotide diversity further highlighted this contrast (Figure 6(C)): while the CDS region maintained a stable baseline diversity, the 3’UTR exhibited a dramatic spike in diversity (π >0.6) immediately upstream of the target site. Crucially, the π value dropped abruptly to zero at the exact location of the *Os-milRNA2* binding site (Figure 6(C)). The alignment coverage analysis (Figure 6(D)) confirms that these zero regions do not indicate extreme sequence conservation; instead, they correspond to a sharp decline in sequence occupancy, dropping from 13 species to only 2–3 species. This quantitative evidence demonstrates that the diversity gap results from extensive lineage-specific deletions in non-host species, signifying a localized loss of homologous sequences rather than functional conservation in this region.

Multiple sequence alignment of 3’UTRs from *attacin* orthologs revealed that the Os-milRNA2 binding site is uniquely present in *T. xiaojinensis* (Figure 6(E)), reflecting a highly specific adaptation of the fungus to its host’s genomic sequence. Notably, despite sharing high genetic identity with *T. xiaojinensis* in mitochondrial markers (COI gene), the closely related congeneric species *Thitarodes* aff. *gonggaensis* completely lacks this target motif due to sequence divergence in this region. This lineage-specific loss abolishes Os-milRNA2 binding, revealing a highly specific susceptibility mechanism shaped by host–parasite coevolution.

## Discussion

Slow-killing parasites face a fundamental evolutionary challenge: they must exploit resources from their hosts while preserving host viability long enough to ensure transmission. Our study reveals that *O. sinensis* resolves this dilemma through an extended phenotype: the employment of a cross-kingdom RNA interference strategy in which a fungal small RNA, Os-milRNA2, modulates host immune activity to enable long-term coexistence. Rather than inducing wholesale immune suppression, this mechanism allows the parasite to modulate host defenses with temporal precision, illuminating a general principle of chronic host–pathogen interactions.

Os-milRNA2 directly targets the antimicrobial peptide gene *attacin-1*, establishing an immune equilibrium that permits fungal persistence without triggering immune collapse. Importantly, immune modulation by Os-milRNA2 is neither constitutive nor absolute; instead, it represents a partial and moderate suppression of host immunity that likely facilitates long-term coexistence. Time-course analyses reveal stage-specific regulation: early infection stages are characterized by elevated *attacin-1* expression and low Os-milRNA2 abundance, whereas a transient surge of Os-milRNA2 during mid-infection coincides with sharp *attacin-1* repression. However, this inverse correlation becomes less pronounced at 120 and 150 dpi, coinciding with the transition from stable coexistence to the mummification stage. During this late phase, fungal development shifts from blastospore proliferation to tissue colonization as host physiological functions decline, resulting in a decrease in the regulatory influence of Os-milRNA2. Although the overall correlation between Os-milRNA2 and *attacin-1* across the entire infection cycle appears weak (Spearman’s *R* = −0.17, *p* = 0.6; Fig S1), this reflects non-linear, temporally restricted regulation rather than a lack of functional coupling. Such dynamics are expected for systems optimized to maintain immune homeostasis rather than binary immune suppression. By suppressing immunity only when fungal clearance is most likely, *O. sinensis* preserves basal host defenses against other environmental threats, thereby stabilizing the host environment required for prolonged parasitism.

The temporal specificity of Os-milRNA2 expression reflects the unique biological constraints of the prolonged *O. sinensis* infection cycle. Successful parasitism over several months requires a balance between evading host clearance and maintaining host viability as a nutrient reservoir. Constitutive immune suppression would likely compromise the host’s ability to resist secondary environmental infections, thereby prematurely terminating the fungal development window. Instead, Os-milRNA2 functions as a stage-specific regulator that peaks at 90 dpi, precisely when the fungal burden is highest and the risk of host-mediated clearance is most acute. By selectively downregulating immune effectors like *attacin-1* during this critical mid-infection phase, the fungus maintains a state of stable coexistence. As the infection progresses toward the mummification stage and the fungus transitions from blastospore proliferation to tissue invasion, the requirement for such precise immunological tuning diminishes, resulting in the observed decline of Os-milRNA2. This temporal expression pattern suggests an optimized regulatory mechanism where immune modulation is intensified only during stages of maximum fungal vulnerability, ensuring the successful completion of the parasite’s complex lifecycle.

Although Attacins are classic antibacterial peptides, their role in fungal defense has been widely documented in Lepidopteran species [31,32]. Transcriptomic studies in *Bombyx mori* have shown that *attacin* genes are among the most robustly induced effectors following infection by the entomopathogenic fungus *Beauveria bassiana* [33]. Functionally, Attacins are crucial for clearing fungal cells from the hemolymph and enhancing host survival [34,35]. Mechanistically, Attacins are known to target actively growing cells by disrupting membrane protein synthesis and altering membrane permeability [36,37]. This likely explains their efficacy against *O. sinensis* blastospores, which proliferate via active budding in the host hemolymph. Consequently, the fungal suppression of *attacin-1* via Os-milRNA2 represents a key adaptation to circumvent this potent host defense.

Beyond *attacin-1*, Os-milRNA2 is predicted to interact with additional host targets (Table S4), including a zinc transporter implicated in immune signaling and antimicrobial activity [38–42]. Zinc also modulates several immune signaling pathways, such as the Toll, Imd, and JAK-STAT pathways [43–46]. Although we confirmed this molecular interaction, transcriptional rescue of the transporter was not detected in vivo (Fig S2), likely reflecting its extremely low basal expression in the fat body compared with other tissues such as the midgut. These observations suggest that Os-milRNA2 may operate through layered regulatory effects, with immune modulation emerging from coordinated, tissue-specific influences rather than reliance on a single dominant target. Among these, *attacin-1* represents a central and evolutionarily conserved effector of insect humoral immunity, making it an ideal focal point for establishing causal links between fungal sRNA delivery and infection outcome.

Our in vivo loss-of-function experiments establish Os-milRNA2 as a modest virulence factor. Antagonizing this sRNA restores *attacin-1* expression and dramatically reduces fungal burden, despite substantial inter-individual variation in immune transcript levels. The consistency of fungal clearance suggests the existence of an immunological threshold: complete restoration of antimicrobial gene expression is not required; rather, elevating immune activity beyond a critical protective level is sufficient to tip the balance in favor of the host. This robustness highlights the resilience of the insect immune system and emphasizes how precisely parasite-mediated suppression must be tuned to maintain chronic infection.

Evolutionary analysis suggests that the Os-milRNA2 - *attacin-1* interaction exemplifies the extraordinary precision and rapid divergence of cross-kingdom RNAi interfaces. By targeting the rapidly diversifying 3’UTR of *attacin-1* in *T. xiaojinensis*, *O. sinensis* can precisely tune host immunity to support persistent colonization without triggering premature death. This molecular match likely underpins the high infection efficiency and robust development observed in the *T. xiaojinensis* system. In contrast, the absence of this target site in *T*. aff. *gonggaensis* indicates that different host lineages may have evolved distinct regulatory interfaces, highlighting sRNA-target matching as a key determinant of host suitability in this chronic infection model.

Conceptually, this interaction is consistent with the extended phenotype [47], in which parasite genes exert functional effects beyond the parasite’s physical boundaries. As suggested by recent host-manipulation genomics [19,20], RNA-mediated interference may be strategically favored over proteinaceous effectors in chronic infection models. Compared to proteins, milRNAs impose a lower metabolic cost and exhibit lower immunogenicity, allowing the fungus to maintain long-term suppression of host immunity (e.g. *attacin-1*) without triggering a fatal hyper-inflammatory response. By delivering Os-milRNA2 into host immune cells, *O. sinensis* integrates its genetic regulation into the host immune network, effectively extending fungal control into host physiology. Rather than dismantling host immunity, the fungus rewires immune logic to maintain a stable, permissive host state until its life cycle transitions to the terminal reproductive phase. This strategy illustrates how chronic parasites optimize fitness by reshaping host regulatory circuits rather than overwhelming them.

Despite these findings, several limitations and open questions remain. Survival analysis was not performed due to the chronic infection cycle and injection-related stress; instead, fungal burden was used as a quantitative indicator of host immunity. Similarly, while specific antibodies for Attacin-1 were unavailable, its functional role is supported by the significant reduction in fungal load following *attacin-1* transcript restoration. Additionally, our evolutionary analysis was limited by the available genomic data for early-diverging lepidopteran lineages. Beyond these experimental constraints, the means by which fungal sRNAs are packaged, protected, and delivered into host cells is unknown, necessitating future studies into whether *O. sinensis* employs extracellular vesicles or RNA-binding proteins [48,49]. Furthermore, population genetics studies are needed to examine how single nucleotide polymorphisms (SNPs) in host 3’UTRs and potentially compensatory mutations in fungal milRNAs drive host-pathogen co-evolution and lineage-specific interactions. Clarifying how Os-milRNA2 interfaces with host Argonaute proteins while accounting for this genetic diversity will further elucidate the molecular infrastructure and evolutionary logic underlying cross-kingdom RNA communication.

More broadly, given the prevalence of chronic infections across biological systems, cross-kingdom sRNA-mediated immune tuning may represent a widespread evolutionary strategy by which parasites reconcile virulence with long-term persistence. Our findings position fungal milRNAs as evolutionarily agile effectors that shape host immunity through rapidly evolving regulatory interfaces, providing insight into how host–pathogen coevolution sculpts the molecular architecture of immune control.

## Conclusion

*Ophiocordyceps sinensis* exploits cross-kingdom RNA interference to modulate host immunity and ensure long-term survival. The fungal small RNA Os-milRNA2 suppresses *attacin-1* expression, permitting persistent infection without premature host death, while its inhibition restores host resistance and limits fungal growth. This interaction bears signatures of precise co-evolution: although attacin orthologs coding sequences are globally conserved, the 3’UTR has undergone rapid diversification. Notably, the Os-milRNA2 target site is uniquely retained in *T. xiaojinensis* but is absent in the closely related host species *Thitarodes* aff. *gonggaensis* and non-host species. This indicates that while *O. sinensis* infects diverse *Thitarodes* species, the Os-milRNA2-mediated suppression mechanism is strictly lineage-specific. Together, these findings uncover a molecular mechanism underlying prolonged parasitism and illustrate how host pathogen co-evolution generates distinct regulatory interfaces across different host lineages.

## Materials and methods

### Sample collection and sRNA sequencing

*Thitarodes xiaojinensis* larvae and *Ophiocordyceps sinensis* isolates were provided by Dongguan HEC Cordyceps R&D Co., Ltd. (Guangdong, China). These larvae originated from a standardized laboratory-reared colony whose ancestral founders were collected from Xiaojin County, Sichuan Province, China. Using an artificially bred population ensured the uniformity of the experimental materials and eliminated the need for wild collection permits. Infected *Thitarodes xiaojinensis* larvae were maintained at 16°C and 75% relative humidity in complete darkness to mimic conventional environmental conditions. Dissect the larvae of *T. xiaojinensis* infected by *O. sinensis* for 3 months (a stage where *O. sinensis* stably coexists with the host and begins extensive proliferation). Rinse the larvae three times with Ring’s reagent to remove *O. sinensis* spores from the hemocoel. Subsequently, collect the fat body tissue and extract total RNA using TRIzol reagent (Invitrogen^TM^, Carlsbad, CA, USA). Confirm the absence of fungal *actin* gene in the total RNA usingRT- qPCR. The absence of fungal contamination was confirmed by RT-qPCR (35 cycles threshold) using *O. sinensis*-specific F-actin capping protein subunit beta gene primers, with no detectable amplification across all tested host samples. The RNA samples were then sent to Beijing Biomarker Technologies Co., Ltd. for small RNA sequencing.

qPCR

Total RNA was extracted from the fat body of *T. xiaojinensis* using TRIzol reagent (Invitrogen, Carlsbad, CA, USA) and reverse transcribed into cDNA using HisyGo RT Red SuperMix for qPCR (+gDNA Wiper) (Vazyme Biotech, Nanjing, China). qPCR was performed using gene-specific primers (Supplementary Table S1) and PerfectStart® Universal Green qPCR SuperMix (TransGen Biotech, Beijing, China). *Tx-RPS3* served as an internal control. The relative expression of *Os-FcpB*, *attacin-1* was calculated using the $2^{-\Delta \Delta C_{T}}$ method.

sRNA data process

Adapters and low-quality sequences from the raw data were removed using Trim-Galore, and sequence quality was assessed using FastQC. Clean reads were then aligned to the *O. sinensis* genome using Bowtie [50] (version 1.2.3) with the following strict parameters: bowtie -v 0 -m 1 –best – strata. These parameters ensure only uniquely mapping reads with no mismatches are reported. Specifically: (*i*) -v 0: permits no mismatches; (*ii*) -m 1: prevents the reporting of reads that map to multiple genomic locations; (*iii*) – best – strata: report only the alignments in the best “stratum,” which are those with the least mismatch. Reads aligning to tRNA and rRNA loci in *O. sinensis* genome were subsequently filtered out. The remaining reads were further filtered to retain only those with lengths between 18 and 30 base pairs (inclusive). Relative abundance was then calculated and normalized to transcripts per million (TPM) using the following formula: TPM = (actual miRNA counts/length)/(total counts of clean reads) ×10^6^.

### Target prediction and functional annotation

A database was constructed using the 1000 bp downstream sequence from stop codon of all genes in the *T. xiaojinensis* genome, extracted with the TBtools [51] GXF sequence extractor. The genome assembly of *T. xiaojinensis* was downloaded from the NCBI GenBank database under the accession number GCA_042246355.1. MilRNAs target were predicted by three software: miranda [52], PITA [53] and RNAhybrid [54]. The following thresholds were applied for each tool: (*i*) miranda: score ≥ 140 (default); (*ii*) PITA: energy ≤ −20; (*iii*) RNAhybrid: parameters -u 0 -e −20 -f 2,8. The specific settings for RNAhybrid were as follows: (a) -u 0: The mismatch ring of both the upper and lower chains cannot contain more than 1 mismatched base; (b) -e −20: The minimum free energy for matching two sequences cannot be less than −20; (c) -f 2,8: The second to eighth bases of miRNA and the target sequence must be perfectly matched. Potential targets were identified as those predicted by all three software packages. The intersection of these predictions is visualized using a Venn diagram (http://hurlab.med.und.edu:3838/VennDetail/). GO and KEGG pathway enrichment analyses were performed using TBtools (v2.103) with the integrated GO and KEGG databases. A hypergeometric test with Benjamini–Hochberg correction for multiple testing (adjusted *p*-value < 0.05) was used.

### In vitro and in vivo validation experiments for effects of Os-milRNA2

Dual-luciferase reporter assay was employed for in vitro verification. MiRNA mimics were synthesized by Suzhou Genewiz Technologies Co., Ltd. The target regions (approximately 400 bp) and corresponding mutant sequences lacking the target site were cloned into the pmirGLO plasmid. MiRNA mimics and plasmids were co-transfected into HEK293T cells using Lipofectamine 3000 (Invitrogen^TM^, Carlsbad, CA, USA) in Opti-MEM (Invitrogen^TM^, Carlsbad, CA, USA). After 48 hours, cells were harvested and lysed. Firefly and *Renilla luciferase* activities were measured using the TransDetect Double-Luciferase Reporter Assay Kit (TransGen Biotech, Beijing, China). Cy3 conjugated Os-milRNA2 (50 μM) was synthesized by GENCEFE Biotech Co., Ltd and injected into the hemocoel of *T. xiaojinensis* at 3 months post infection, after 24 h post injection, the fat body was dissected and washed with PBS, fixed in 4% paraformaldehyde, followed by nucleic acid staining with DAPI. Images were acquired by fluorescence microscope.

### In vivo inhibition assay of Os-milRNA2

To investigate the in vivo function of Os-milRNA2, a specific Os-milRNA2 inhibitor was injected into the hemocoel of *T. xiaojinensis* larvae that had been infected by *O. sinensis* for 3 months. A commercially available universal non-targeting antagomir (Antagomir-NC) served as the negative control. Both oligonucleotides were chemically synthesized by GENCEFE Biotech Co., Ltd. (China). The Antagomir-NC sequence is 5”-CAGUACUUUUGUGUAGUACAAA-3.‘ To ensure in vivo stability and membrane permeability, the oligonucleotide incorporated the following chemical modifications: (1) full-length 2’-O-methyl (2‘−OMe) modifications; (2) phosphorothioate linkages at the first two (5’−end) and last four (3”−end) nucleotides; and (3) a 3’-cholesterol conjugation. The Antagomir-NC sequence was verified to share no significant homology with either the *T. xiaojinensis* host or *O. sinensis* fungal genomes (Supplementary Table S6). Injections were administered weekly for four consecutive weeks. One week after the final injection, hemolymph was harvested to quantify *O. sinensis* blastospores, and fat body tissues were collected to determine *attacin-1* gene expression levels.

### Sequence retrieval and alignment

The 3’ Untranslated Region (3’UTR) sequences of *attacin-1* orthologs were retrieved from the NCBI Nucleotide database. The dataset included the host species *Thitarodes xiaojinensis* and representative non-host species from the order Lepidoptera (e.g. *Bombyx mori*, *Ostrinia furnacalis*, *Manduca sexta*, *Helicoverpa armigera*, etc.). Distantly related orders (e.g. Diptera) were excluded to minimize alignment artifacts caused by sequence saturation. Multiple sequence alignment was performed using the ClustalW algorithm implemented in MEGA11 [55] (Molecular Evolutionary Genetics Analysis, version 11) with default parameters.

### Binding affinity-based target prediction

The binding affinity between the fungal sRNA (Os-milRNA2) and the *attacin-1* 3’UTR sequences was predicted using miRanda [52]. Minimum Free Energy (ΔG) values were calculated to assess thermodynamic stability. A strict score threshold (>140) was applied to filter nonspecific interactions.

### Phylogenetic analysis

Phylogenetic analyses were performed using MEGA 11 [55]. Sequence alignments were generated using the ClustalW algorithm, specifically, CDS sequences were aligned by codons to preserve the reading frame, while 3’UTR sequences were aligned as nucleotides. The best-fit nucleotide substitution models were determined based on the Bayesian Information Criterion (BIC). Maximum Likelihood (ML) trees were constructed with 1000 bootstrap replications to assess nodal support, utilizing the Partial Deletion method for gap treatment. For the CDS tree, the General Time Reversible model with Gamma distribution and Invariant sites (GTR+G+I) was applied with a site coverage cutoff of 95%. In contrast, the 3’UTR tree was inferred using the Tamura 3-parameter model with Gamma distributed rate heterogeneity (T92+G) and a site coverage cutoff of 80%. To reflect the evolutionary divergence between Exoporia and Ditrysia, both trees were rooted using *Thitarodes xiaojinensis* as the outgroup.

### Nucleotide diversity (π) calculation

Multiple sequence alignment of the *attacin-1* gene was performed using MEGA 11 [55], followed by manual inspection to ensure alignment accuracy. The 3’UTR region was extracted for subsequent analysis. Nucleotide diversity [56] (π) was calculated using DnaSP v6 [57] to assess the degree of genetic polymorphism. Notably, all alignment positions were retained in the analysis. No sites containing gaps or missing data were excluded, in order to fully capture the genetic information preserved within regions containing insertions or deletions.

### Target site visualization

Genomic data for *attacin-1* 3” UTRs were derived from downloaded genome (*T. xiaojinensis*), chromosome walking (*T*. aff. *gonggaensis*), or the NCBI database (other species), with all 3” UTR fragments manually curated. To illustrate the molecular divergence at the sRNA binding site, the aligned sequences flanking the target region were extracted and rendered using N·ESPript [58] 0.9 (https://nespript.ibcp.fr/ESPript/NESPript/index.php). The similarity calculation was set to %Equivalent mode. Conservation of upstream motifs vs the specific loss of the seed-matching region in non-host species was highlighted to demonstrate host-specific targeting.

### New miRNA prediction

Novel miRNAs were predicted using miRDeep2 [59]. Following genome mapping and precursor identification, miRDeep2 compares predicted mature miRNA sequences against known miRNAs in miRBase. A miRNA is classified as novel if no significant sequence similarity is found within the database. miRDeep2 employs a scoring system based on read abundance, precursor structural features, and star sequence presence, with higher scores indicating greater prediction confidence. Novel miRNAs are scored and ranked alongside known miRNAs.

### sRNA RT-qPCR

The miRNA was extracted from each sample using EasyPure miRNA Kit (TransGen Biotech, Beijing, China) and reverse transcribed to complementary DNA (cDNA) using TransScript miRNA First-strand cDNA Synthesis SuperMix (TransGen Biotech), and qRT-PCR reactions were performed using PerfectStart Green qPCR SuperMix (TransGen Biotech, Beijing, China) according to the manufacturer’s protocol. Tx-U6 served as an internal control. The relative expression of milRNAs were calculated using the $2^{-\Delta \Delta C_{T}}$ method.

### Statistical analysis

Data are presented as mean ± standard error of the mean (SEM) from at least three independent biological replicates. Statistical analyses were performed using two-tailed unpaired Student’s t-tests for two-group comparisons and one-way ANOVA for multiple-group comparisons. A *p*-value of less than 0.05 was considered statistically significant.

## Supplementary Material

Table_S3.xlsx

Fig_S2.tif

Table_S2.xlsx

Table_S5.xlsx

supplementary_figure_and_table_legend.docx

Fig_S1.tif

Table_S6.xlsx

Table_S1.docx

Table_S4.xlsx

## Data Availability

The raw omics data that support the findings of this study are openly available in the Genome Sequence Archive [63] in National Genomics Data Center [64], China National Center for Bioinformation / Beijing Institute of Genomics, Chinese Academy of Sciences at https://ngdc.cncb.ac.cn/gsa/browse/CRA038699. The source data underlying all figures and the Supplementary Tables are openly available in the Science Data Bank (ScienceDB) at https://doi.org/10.57760/sciencedb.31251 [65].
