# Supplementary material for: A pathogen-derived small RNA modulates host immunity for chronic parasitism
Source: Virulence. 2026 Nov 9;17(1):2757924. doi: 10.1080/21505594.2026.2757924 (PMC13757692; doi:10.1080/21505594.2026.2757924)
Supplement: Supplementary file 1 [file KVIR_A_2757924_SM7249.docx]

Table S1. List of primer sequences used for quantitative real-time PCR (qPCR) analysis.

The table provides the names and 5' to 3' sequences of primers used for the host genes and pathogen-derived milRNAs in this study. F: forward primer; R: reverse primer.

| primer | sequence |
| --- | --- |
| q-*Os-FcpB*-F | AGTTGCCGCCCTCGTAGTAGAC |
| q-*Os-FcpB*-R | TCCTTCTGTCCCATGCCAACCA |
| q-RPS3-F | TTATTCCGGCGTTGAGGTCC |
| q-RPS3-R | CTGATACGACGGCCCTTCTC |
| q-*attacin-1*-F | CCGCCGCTAAAGTGAACCTGAT |
| q-*attacin-1*-R | CGTTGCCACCGTTGGTCTTGA |
| q-zinc-F | TCAGACTCATCAGGCAAGG |
| q-zinc-R | GCGTGTTCGGTGACAATG |
| q-Os-milRNA2 | TAATACGTTAGACGTCTCCGATGG |
