# Supplementary material for: A pathogen-derived small RNA modulates host immunity for chronic parasitism
Source: Virulence. 2026 Nov 9;17(1):2757924. doi: 10.1080/21505594.2026.2757924 (PMC13757692; doi:10.1080/21505594.2026.2757924)
Supplement: Supplementary file 2 [file KVIR_A_2757924_SM7252.docx]

**Supplementary Figure and Table Legends**

**Fig. S1. Correlation between Os-milRNA2 and *attacin-1* expression.** Scatter plot illustrating the relationship between relative expression levels of Os-milRNA2 and Log10-transformed *attacin-1* at various time points (60, 90, 120, and 150 dpi). Each colored dot represents an individual sample. The dashed line indicates the linear regression fit. Pearson correlation coefficient (*R*) and *p*-value are shown in the upper left.

**Fig. S2. Validation of zinc transporter as a direct target of Os-milRNA2.** (A) Sequence alignment of Os-milRNA2 and the predicted target site in the zinc transporter mRNA, along with the mutated sequence (zinc transporter-mut). (B) Dual-luciferase reporter assay confirming the interaction between Os-milRNA2 and the zinc transporter target sequence. NC, negative control. (C) Relative mRNA expression of zinc transporter at indicated days post-infection (dpi) adjusted to *RPS3*. Data are presented as mean ± SEM (*n*=3). **p* < 0.05; ****p* < 0.001; ns, not significant (Student’s t-test or One-way ANOVA).

**Table S1. List of primer sequences used for quantitative real-time PCR (qPCR) analysis.** The table provides the names and 5' to 3' sequences of primers used for the host genes and pathogen-derived milRNAs in this study. F: forward primer; R: reverse primer.

**Table S2. Genome mapping analysis of identified Os-milRNAs.** Summary of mapping parameters for Os-milRNAs aligned against the *Ophiocordyceps sinensis* and host (*Thitarodes xiaojinensis*) genomes. The data demonstrate that these small RNAs originate from the *O. sinensis* genome (top panel) and show no significant mapping to the host genome (bottom panel). Flag values: 0, mapped to the forward strand; 16, mapped to the reverse strand; 4, unmapped. Chr, chromosome; CIGAR, Concise Idiosyncratic Gapped Alignment Report.

**Table S3. Functional annotations of predicted target genes for Os-milRNA1.** This table lists the potential target genes of Os-milRNA1 and their functional annotations retrieved from Pfam, SwissProt, and eggNOG databases. ID, gene model identifier; Pfam, protein family database; SwissProt, manually curated protein sequence database; eggNOG, evolutionary genealogy of genes: Non-supervised Orthologous Groups.

**Table S4. Functional annotations of predicted target genes for Os-milRNA2.** This table lists the potential target genes of Os-milRNA2 and their functional annotations retrieved from Pfam, SwissProt, and eggNOG databases. ID, gene model identifier; Pfam, protein family database; SwissProt, manually curated protein sequence database; eggNOG, evolutionary genealogy of genes: Non-supervised Orthologous Groups.

**Table S5. miRanda-based binding affinity prediction of Os-milRNA2 binding sites across various insect species.** This table lists the miRanda scores and minimum free energy (ΔG) in 12 lepidopteran species and *Drosophila melanogaster*. Host status indicates the ecological relationship of each species to *O. sinensis*. ΔG, Gibbs free energy; kcal/mol, kilocalories per mole.

**Table S6. In silico specificity analysis of Antagomir and NC sequences.** This table summarizes the BLAST search results of Antagomir and Negative Control (NC, cel-miR-39-3p) sequences against the *T. xiaojinensis* and *O. sinensis* genomic databases. WGS, whole genome shotgun; nt, nucleotide collection.
